# Molecular Characterization of Primary Ovarian Angiosarcoma in a Patient With a History of Mature Ovarian Teratoma

**DOI:** 10.7759/cureus.95651

**Published:** 2025-10-29

**Authors:** Zarrin Hossein-Zadeh, Divya Gowthaman, Tahyna Hernandez, Tony Philip, Marina Frimer

**Affiliations:** 1 Pathology, Northwell Health, Greenvale, USA; 2 Obstetrics and Gynaecology, Northwell Health, New York, USA; 3 Pathology and Laboratory Medicine, Northwell Health, Greenvale, USA; 4 Gynecologic Oncology, Northwell Health, New Hyde Park, USA

**Keywords:** angiosarcoma of the ovary, mature cystic teratoma of the ovary, next-generation sequencing in ovarian malignancies, primary germ cell neoplasm of the ovary, primary somatic malignancy of the ovary

## Abstract

Angiosarcomas of the female reproductive tract are rare, most commonly occurring in the uterine body, and especially rare in the ovaries. Furthermore, the concomitance of angiosarcomas and mature cystic teratomas of the same ovary is even more sparse. We report a case of a 34-year-old G2P2 female with angiosarcoma of the left ovary, approximately 12 years after an initial diagnosis of mature cystic teratoma of bilateral ovaries with molecular and next-generation sequencing (NGS) studies supporting the diagnoses of two distinct primaries. Our case is unique as comparative NGS and copy-number mutation studies established that the two lesions are not genetically related, favoring distinct tumors that may be helpful in guiding therapeutic management.

## Introduction

Ovarian mature cystic teratomas are the most common germ cell tumors, accounting for 27-44% of ovarian neoplasms typically in women of reproductive age [1]. Their components, which derive from ectoderm, endoderm, and mesoderm, may undergo a malignant transformation in 0.2-2% of cases [2]. Risk factors for malignant transformation of teratomas include age over 45 years, postmenopausal status, elevated CA 125, and tumor size greater than 10 cm [1]. The most common neoplasm found within the mature cystic teratoma is squamous cell carcinoma [3]. Other tumors include basal cell carcinoma, melanoma, adenocarcinoma, sarcoma, thyroid carcinoma, and angiosarcoma. The behavior of malignancies in mature cystic teratomas is determined by their phenotype and not their derivation from germ cells [3].

Sarcomas, particularly angiosarcomas of the female genital tract are very rare and comprise less than 1% of all ovarian malignancies [4]. Ovarian angiosarcoma was first described in 1931, and only a few tens of cases have been published in the literature [5]. Angiosarcomas are aggressive tumors with a median survival of 15-30 months [6]. Given the abundant anastomotic channels within angiosarcomas, these tumors are prone to recurrence and metastasis, with an overall poor prognosis [7]. Angiosarcomas are genetically heterogeneous, and most tumors harbor complex karyotypes including trisomy of the 5th chromosome and loss of Y chromosome, KDR (knockdown resistance) mutations, PTPRB (gene encoding protein tyrosine phosphatase, receptor type B), PLCG1 (gene encoding phospholipase C gamma 1), and a novel fusion gene in the NUP160-SLC43A3 [5].

To date, there are less than 40 cases of ovarian angiosarcomas reported in the literature, of which less than 1% is thought to arise from previous malignant transformation of mature cystic teratoma. Most of the reported cases of somatic transformation are based on the presence of a history or concurrent mature cystic teratoma and a somatic malignancy; however, few studies have actual documented molecular evidence to link the two. Our case is the first to describe an angiosarcoma of the ovary, in a patient with previous history of bilateral mature cystic teratomas, and comparative next-generation sequencing (NGS)/copy-number profile studies supporting the diagnoses of distinct primaries.

## Case presentation

The patient is a 34-year-old female with a history of prior cesarean section, laparoscopic right salpingo-oophorectomy, and partial left salpingo-oophorectomy with a diagnosis of bilateral mature cystic teratomas, who presented to the emergency room with shortness of breath for 10 days and multiple episodes of nausea and vomiting. The patient underwent imaging studies.

**Figure 1:**
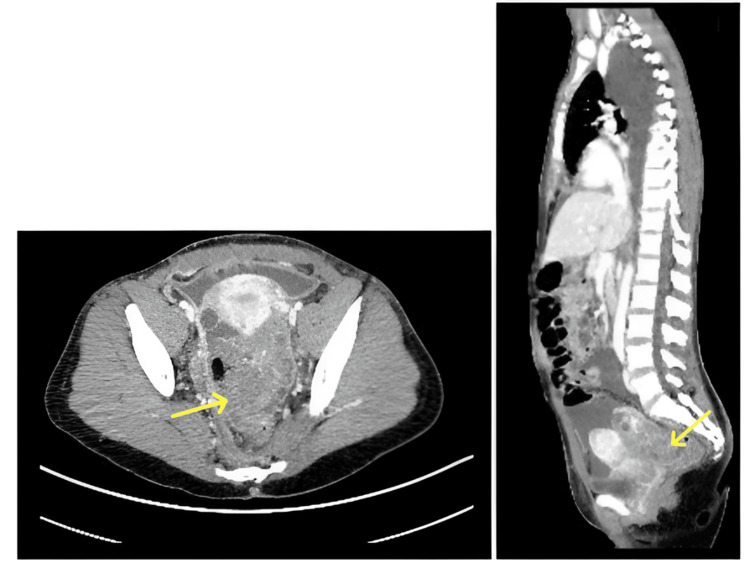
Preoperative CT image Preoperative CT showing a 6.4 cm x 8.2 cm predominantly soft tissue density lesion (denoted by a yellow arrow) with a small fatty component in the cul-de-sac.

Computerized tomography of the chest revealed large right and moderate left pleural effusions with near total opacification of the right lower lobe. A right thoracentesis was performed with 1570 ml of serosanguinous fluid removed; however, cytology was found to be negative for malignancy. A chest tube was placed for rapid re-accumulation of the pleural effusion. CT of the abdomen and pelvis showed a 6.4 x 8.2 cm predominantly soft tissue density lesion in the cul-de-sac with large intra-abdominal ascites (Figure 1). A paracentesis was then performed, revealing negative pathology for malignancy.

She then underwent an exploratory laparotomy, left salpingo-oophorectomy, and peritoneal biopsies with intraoperative frozen section concerning for lymphoma. Intraoperatively, an 8 cm irregular friable complex appearing mass was noted to be originating from the left ovary. The right ovary was absent because of prior surgery. The uterus and left fallopian tube were grossly normal. Flimsy ill-defined red lesions were seen throughout the peritoneum. Her immediate postoperative course was uncomplicated. The chest tube was removed with resolution of the right effusion, and the patient was discharged on postoperative day 2 with plan for outpatient follow-up with gynecologic oncology and medical oncology. Histopathologic and immunohistochemical staining confirmed the presence of an ovarian angiosarcoma.

On postoperative day 13, she was seen by outpatient gynecologic oncologist and endorsed progressive shortness of breath. She was found to be tachycardic with the heart rate increased up to 120 bpm with decreased breath sounds bilaterally. The patient underwent a right video-assisted thoracoscopic surgery (VATS) with right PleurX placement, pleural biopsy, and left pigtail catheter placement by cardiothoracic surgery. Histopathology of the right pleura was positive for metastatic angiosarcoma. Medical oncology advised doxorubicin and ifosfamide regiment, and the patient was discharged after completion of the first cycle. She received three cycles of doxorubicin and ifosfamide in total; however, ifosfamide had to be discontinued due to Grade 2 (moderate) neurotoxicity. She proceeded with single-agent doxorubicin for a total of seven cycles.

Pathologic findings

Gross Descriptions

**Figure 2:**
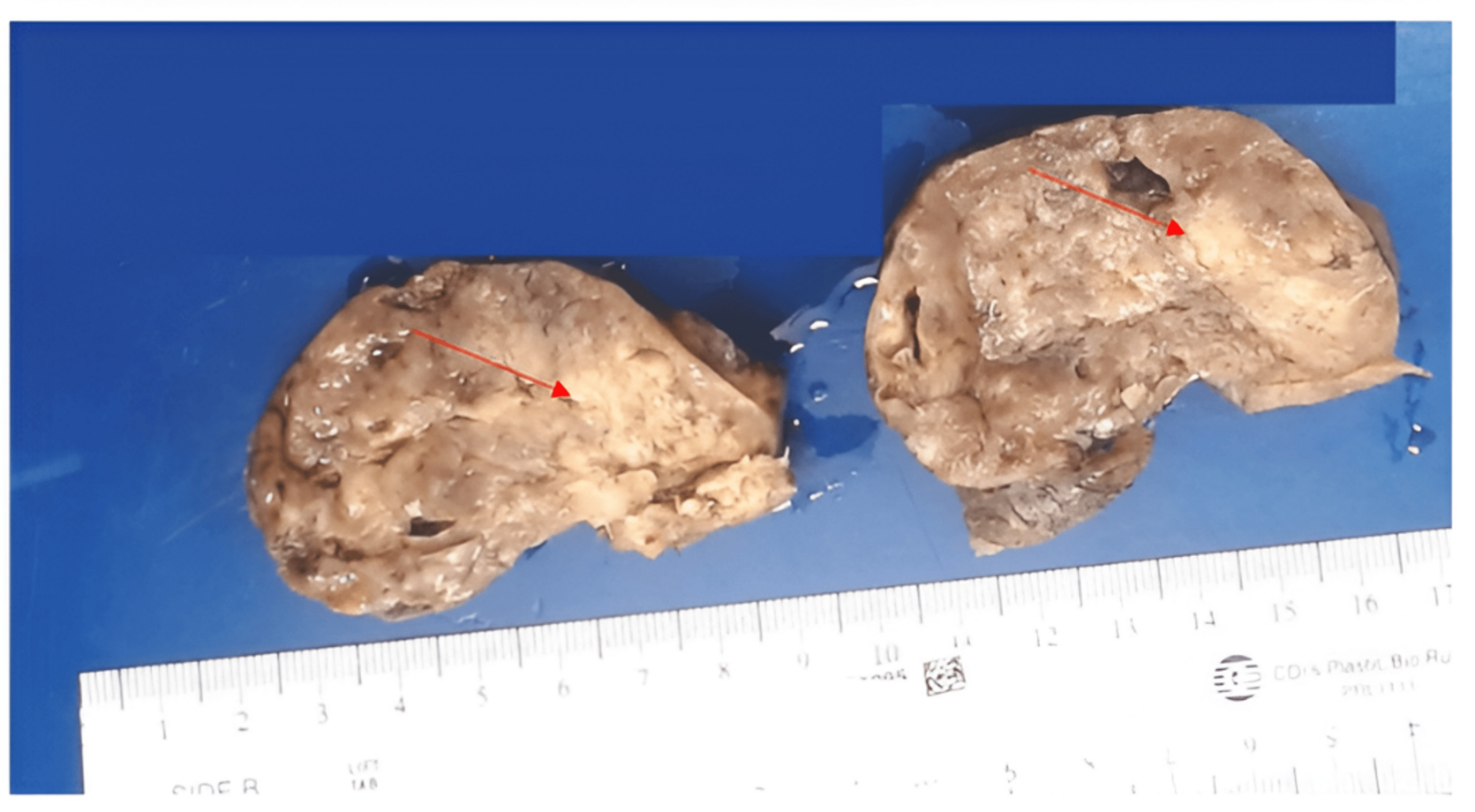
Gross image of the left ovary Gross image of the left ovary demonstrating a 2 cm tan and fleshy mass, as depicted by the arrow.

The Department of Pathology received a 6.6 x 6.5 x 4.3 cm, 138-gram ovary, with a smooth and tan cortical surface (Figure 2). The cut surface of the ovary showed a 2 cm tan and fleshy mass. On the cut section, the tumor was cystic and solid with fleshy, gelatinous areas and necrosis. Of note, there was no hair, sebaceous material, or adipose tissue identified.

Microscopic findings

**Figure 3:**
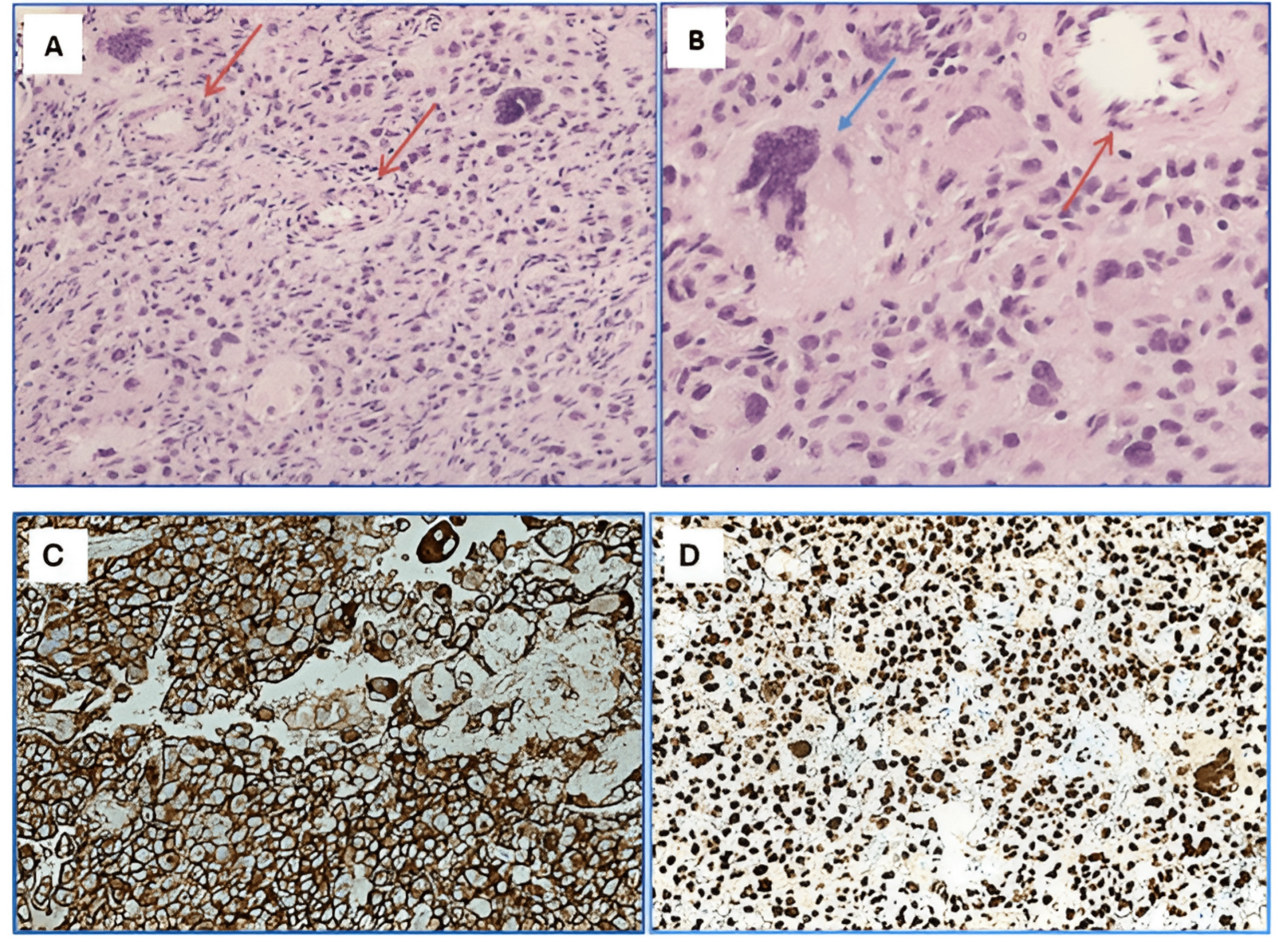
H&E and Immunohistochemical staining A) H&E section at 20x magnification, showing a cellular lesion with atypical cells and numerous vessels (red arrows). B) H&E sections at 40x magnification, showing bizzare cells with irregular nuclei (blue arrow) and a vascular lumen (red arrow). C) CD31 immunostain of ovarian angiosarcoma showing strong CD31 (an endothelial marker) expression at 20x magnification. D) ERG immunostain of ovarian angiosarcoma showing strong ERG (vascular marker) expression at 20x magnification.

Histological sections demonstrated a highly cellular neoplasm involving the ovary, composed of sheets of neoplastic cells with abundant, eosinophilic cytoplasm, and high-grade nuclear atypia including nuclear pleomorphism, multinucleation, and brisk mitotic activity (Figure 3). The tumor was extensively sampled and showed same morphology throughout without areas of well differentiation or teratomatous component.

Differential diagnoses included ovarian germ cell tumors including choriocarcinoma, dysgerminoma, and/or embryonal carcinoma, as well as poorly differentiated sarcomas, in addition to malignant melanoma. An expansile immunohistochemical panel was performed, and the tumor cells were diffusely and strongly positive for CD31, ERG, and D2-40. Tumor cells were negative or weakly positive for all other markers including epithelial, germ cell, smooth muscle, melanocytic, and neural antibodies, ruling out other differentials initially entertained. The morphologic and immunohistochemical features are consistent with ovarian angiosarcoma.

Molecular analysis

Paraffin-embedded blocks from the original left ovarian mature cystic teratoma and the more recent ovarian angiosarcoma samples were sent to Foundation Medicine and FoundationOne CDX (an FDA-approved, tissue-based, comprehensive genomic profiling test for all solid tumors) for RNA and Companion Diagnostic (CDx) NGS. A mutation in the EGFR-V323I was identified in the original mature cystic teratoma of the ovary. Molecular findings for the left ovarian angiosarcoma include the following aberrancies: BRIP1 rearrangement exon7, CRKL amplification, MAPK1 amplification, and TP53 loss. The NGS interpretive report of the manual review of the copy-number profiles from prior left mature cystic teratoma of the ovary, and the more recent left ovarian angiosarcoma showed that the tumors do not share overlapping copy-number profiles. This finding, in conjunction with a lack of shared reportable alterations, suggests that the angiosarcoma in this case and the patient's sequenced prior teratoma are clonally unrelated and thus represent distinct tumors.

## Discussion

Ovarian angiosarcoma is extremely uncommon, and less than a few tens of cases have been documented to date, predominantly affecting premenopausal women aged less than 50 years [8]. It can occur alone or in conjunction with teratomas, mucinous cystadenocarcinoma, and dermoid cysts among other ovarian tumors [9]. The clinical manifestations of primary versus secondary/metastatic ovarian angiosarcoma lack specificity and can include abdominal pain and unexplained gastrointestinal or urinary symptoms [10]. On histopathology, primary or secondary ovarian angiosarcoma consists of endothelial cells with varying degrees of atypia. In well-differentiated cases, the tumor cells may recapitulate vascular structures; however, in poorly differentiated cases, discohesive single cells with highly pleomorphic and bizarre atypical cells can be observed [11]. Given the rarity of this condition, there are not many reported cases in the literature of ovarian angiosarcoma, particularly in patients with a previous history of cystic teratoma in the same ovary. Nonetheless one essential question is whether the somatic malignancy arose as the result of neoplastic transformation of the germ cell tumor or due to a de novo process. By immunohistochemistry, the most common markers expressed in ovarian angiosarcoma, primary and secondary, include CD31 (100%), CD34 (98%), vimentin (100%), and factor VIII-related gene (85.7%) [12].

In an extensive review of the literature, Rehman et al. performed a review of patients with ovarian angiosarcoma based on symptoms at presentation, histopathology, therapeutic regimen, and the clinical status of the patients. The histopathology that were assessed and analyzed included the presence of necrosis, hemorrhage, pleomorphism, spindle-shaped cells, epithelioid-shaped cells, mitotic figures, anastomosing vascular channels, and immunohistochemical marker profile. Their findings demonstrated that tumors with necrosis and epithelioid morphology on histology had worse survival outcomes with a median survival of seven months versus 22 months for patients without necrosis. Their survival analysis showed that chemotherapy alone and chemotherapy combined with surgical resection demonstrated prolonged overall survival [13].

Conventional cytogenetic techniques including electrophoretic banding patterns, enzyme polymorphisms, and centromeric hetermorphisms and more recent molecular approaches such as NGS have been utilized in further characterization of germ cell and non-germ cell neoplasms of the ovary [14]. The majority of ovarian mature teratomas develop from ovarian oocytes after completion of meiosis I with failure of meiosis II, therefore containing a homozygous chromosome set that is genetically distinct from the diploid heterozygous genome in somatic cells. However, an estimated 35% of ovarian mature teratomas may arise as a result of meiosis I failure or mitotic division of premeiotic germ cells, consequently inheriting a heterozygous biallelic genome similar to that of the somatic cells [15]. In one study, 63% of mature teratomas displayed partial or complete homozygosity, with the remaining 37% showing heterozygosity. To date, there are not many reported cases in the literature with comparative molecular studies between the primary germ cell and secondary somatic neoplasms of the ovary to distinguish genetic linkage.

In the current case, NGS studies showed the presence of EGFR-V323I mutation in the original mature cystic teratoma, while revealing BRIP1 rearrangement, CRKL amplification, MAPK1 amplification, and TP53 loss in the secondary angiosarcoma. In addition, a manual review of the copy-number profiles from the two lesions showed that the tumors do not share overlapping copy-number profile findings. Our case is unique in reporting comparative molecular findings, which favor the presence of two distinct and genetically unrelated tumors. 

Although NGS testing has revolutionized genomics, it still has limitations, which include some challenges with accurately sequencing repetitive or GC-rich regions, leading to uneven coverage and potential gaps. Short read lengths can hinder the resolution of complex structural variants and haplotype phasing. Due to the massive amount of data generated by the NGS database, the data also may contain sequencing errors, requiring sophisticated bioinformatics for accurate interpretation [14].

Future prospects

Current advancements in molecular techniques, including NGS and comprehensive genomic profiling (CGP) assays, enable panels as such to be incorporated in cases of angiosarcoma or other somatic malignancies, particularly in patients with a known history of germ cell neoplasm. This will allow clinicians to then decipher if the two entities share common mutations or appear to be different clonal populations.

## Conclusions

Angiosarcoma of the ovary is a rare and highly aggressive tumor. Clinically, it presents with abdominal pain and distension. Histopathology and immunohistochemistry are essential for a definitive diagnosis. To date, there are only a few cases of ovarian cystic teratoma and angiosarcoma reported in the literature; however, our case is unique in reporting comparative molecular studies between the primary germ cell mature teratoma and the secondary angiosarcoma. Comparative NGS and copy-number mutation studies established that the two lesions are not genetically related, favoring distinct tumors that may be helpful in guiding therapeutic management. Incorporation of molecular techniques including NGS and comprehensive genomic profiling assays may be paramount in the further characterization of primary and secondary ovarian neoplasms due to therapeutic implications.
